# Evaluation of a clinical decision support tool for osteoporosis disease management: protocol for an interrupted time series design

**DOI:** 10.1186/1748-5908-6-77

**Published:** 2011-07-22

**Authors:** Monika Kastner, Anna Sawka, Kevin Thorpe, Mark Chignel, Christine Marquez, David Newton, Sharon E Straus

**Affiliations:** 1 Department of Health Policy, Management and Evaluation, University of Toronto, Toronto, Ontario, Canada; 2 Division of Endocrinology, University Health Network and University of Toronto, Toronto, Ontario, Canada; 3 Dalla Lana School of Public Health, University of Toronto, Toronto, Ontario, Canada; 4 Department of Mechanical and Industrial Engineering, University of Toronto, Toronto, Ontario, Canada; 5 Li Ka Shing Knowledge Institute of St. Michael's Hospital, Toronto, Ontario, Canada; 6 Faculty of Medicine, University of Toronto, Toronto, Ontario, Canada

## Abstract

**Background:**

Osteoporosis affects over 200 million people worldwide at a high cost to healthcare systems. Although guidelines on assessing and managing osteoporosis are available, many patients are not receiving appropriate diagnostic testing or treatment. Findings from a systematic review of osteoporosis interventions, a series of mixed-methods studies, and advice from experts in osteoporosis and human-factors engineering were used collectively to develop a multicomponent tool (targeted to family physicians and patients at risk for osteoporosis) that may support clinical decision making in osteoporosis disease management at the point of care.

**Methods:**

A three-phased approach will be used to evaluate the osteoporosis tool. In phase 1, the tool will be implemented in three family practices. It will involve ensuring optimal functioning of the tool while minimizing disruption to usual practice. In phase 2, the tool will be pilot tested in a quasi-experimental interrupted time series (ITS) design to determine if it can improve osteoporosis disease management at the point of care. Phase 3 will involve conducting a qualitative postintervention follow-up study to better understand participants' experiences and perceived utility of the tool and readiness to adopt the tool at the point of care.

**Discussion:**

The osteoporosis tool has the potential to make several contributions to the development and evaluation of complex, chronic disease interventions, such as the inclusion of an implementation strategy prior to conducting an evaluation study. Anticipated benefits of the tool may be to increase awareness for patients about osteoporosis and its associated risks and provide an opportunity to discuss a management plan with their physician, which may all facilitate patient self-management.

## Background

There are over 200 million people worldwide who have osteoporosis, representing a considerable healthcare and financial burden [1-5]. The disease burden will be further compounded by an increasingly aging population, which will likely lead to more people who will suffer from osteoporosis [2,3,6]. The clinical consequence of osteoporosis is fragility fractures; vertebral and hip fractures have the most devastating prognosis [7] and are associated with an increased risk of death [8]. Furthermore, these fractures can significantly impair quality of life, physical function, and social interaction and can lead to admission to long-term care [9-11]. Although guidelines are available for osteoporosis disease management [12-14], many patients are not receiving appropriate diagnostic testing or treatment [15-19]. Clinical decision support systems (CDSSs) may be one solution to closing these practice gaps because they can provide evidence at the point of care to facilitate disease management. CDSSs work by generating patient-specific assessments or recommendations for clinicians; software algorithms match pieces of information from a knowledge database to relevant clinical data [20-22].

To determine what features of osteoporosis tools support clinical decision making in osteoporosis disease management, we conducted a systematic review of randomized controlled trials [23]. Findings showed that interventions consisting of reminders and education targeted to both physicians and patients were more promising for increasing osteoporosis investigations and treatment than single-component or single-target interventions [23]. We first developed a conceptual design for an osteoporosis disease-management tool using these findings and input from clinicians and experts in information technology and human-factors engineering. We then built a prototype using findings from a qualitative study of focus groups with family physicians [24]. The prototype was further refined in a series of usability studies with its target end users (physicians and patients at risk for osteoporosis) [25]. The osteoporosis tool is targeted to family physicians, and patients at risk for osteoporosis (women age ≥50 years, men age ≥65 years) and consists of three components: (1) a short (three to five minutes) electronic risk assessment questionnaire (RAQ) targeted to at-risk patients to be completed on a touch-screen tablet PC in the clinic examination room (while they wait for their physician); (2) a one-page best practice recommendation prompt (BestPROMPT) outlining appropriate osteoporosis disease-management recommendations (*e.g.*, to initiate bone mineral density [BMD] testing and osteoporosis treatment) customized according to patients' RAQ responses and available to physicians in the few minutes before the visit; (3) and a one-page, customized osteoporosis education (COPE) sheet tailored to patients' RAQ responses and given at the end of their physician visit. The functional osteoporosis tool is accessible online at http://knowledgetranslation.ca/osteo_final/index.html.

The objectives of the current study are to implement the osteoporosis tool prototype in three family practice settings and to conduct a pilot evaluation study to test the impact of the osteoporosis tool on disease management (*i.e.*, appropriate initiation of osteoporosis investigations and medications) using the quasi-experimental ITS design. Specifically, we will answer the following questions: (1) Does use of an osteoporosis disease-management tool by family physicians lead to enhanced osteoporosis management according to current clinical practice guidelines, as measured by increased BMD testing and prescription of osteoporosis medications such as bisphosphonates?; (2) How do clinicians perceive the utility of the tool for changing clinical practice and knowledge uptake?; (3) What is the impact of the tool on clinician adoption and satisfaction with the tool?; (4) Do family physicians use the tool in similar ways across different practice settings (*e.g.*, solo practice vs. group practice)?

## Methods

**Figure 1:**
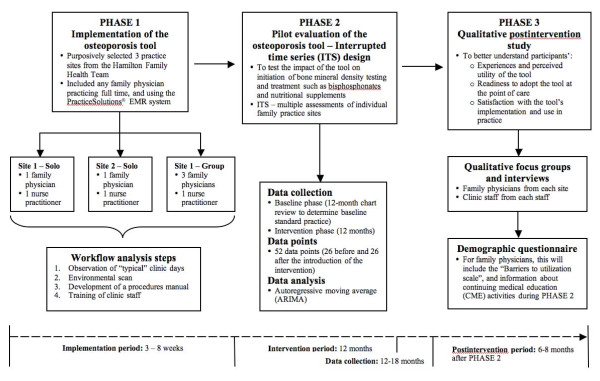
**The osteoporosis tool will be evaluated according to a three-phase process**: implementation of the osteoporosis prototype in three family practices (phase 1), evaluation of the tool using an ITS design (phase 2), and a qualitative evaluation to identify the barriers to using the tool in practice (phase 3).

The osteoporosis tool will be evaluated according to a three-phase process (see Figure 1): implementation of the osteoporosis prototype in three family practices (phase 1), evaluation of the tool using an ITS design (phase 2), and a qualitative evaluation to identify the barriers to using the tool in practice (phase 3).

### Phase 1: implementation of the osteoporosis tool

The tool will be implemented in three family practice settings selected purposively from the Hamilton Family Health Team (FHT). This is the largest of the 150 approved primary care FHTs in Ontario, Canada, serving approximately 250,000 people [26]. It includes a comprehensive team of healthcare professionals, including 129 family physicians, 114 nurses and nurse practitioners, 20 registered dieticians, 77 mental health counsellors, 22 psychiatrists, and 7 pharmacists. A unique feature of this FHT is that all physicians use an electronic patient record system, although not every physician uses the same system [26]. We purposively selected three family practices (two solo and one group practice) that used the same electronic medical record (EMR) system (*i.e.*, PracticeSolutions^® ^[Practice Solutions, Ottawa, Ontario, Canada]) to facilitate implementation of the osteoporosis tool and subsequent data collection during the evaluation study.

To ensure optimal functioning of the prototype and to minimize disruption to usual practice, the osteoporosis tool will be tailored to the practice and workflow of each practice setting. We will complete a workflow analysis in these family practice settings to ensure the prototype's optimal functioning, minimize the disruption of the tool on usual practice, and determine if the tool could be used by patients and physicians at the point of care. Our previous work revealed that workflow differences needs to be considered during the tool design process [24,25], particularly for complex interventions that are delivered at the point of care.

First, we will perform a workflow analysis, which will include observation of clinic staff during "typical" clinic days. Two researchers will document the patient registration process (particularly how patients are moved from the waiting area to the examination room) and estimate the average time that patients wait for their physician, the length of patient visits, and time between visits. Second, researchers and an information technologist will conduct an environmental scan to ensure appropriate equipment installation. Third, a procedures manual will be developed and customized for each site. Lastly, clinic staff (including physicians, nurses, and receptionists) will be trained on how to use and troubleshoot the osteoporosis tool. Once programming is completed and equipment installed, clinics will be instructed to begin using the tool, and observed to correct unanticipated installation, programming, or workflow interruption problems. We will consider the tool as implemented when no new problems are reported for at least one week.

### Phase 2: pilot evaluation study

#### Study design

The osteoporosis tool will be evaluated in a pilot study using the quasi-experimental ITS design. Quasi-experimental designs such as the ITS are particularly strong alternatives to randomized controlled trials (RCTs) [27] and are considered a useful and pragmatic tool, particularly for pilot studies where initial evaluations of interventions and their refinement need to be done before the testing of the tool on a wider scale is justified [27,28]. Results from ITS studies can serve to inform the investigation of mediating factors (for example, if the intervention is found to be more effective at one site but not at another), and they allow for the statistical investigation of potential biases in the estimate of the effect of the intervention [27,28]. For example, this design can address secular trends (*i.e.*, the outcome may be increasing or decreasing over time), history (*i.e.*, there may be trends or seasonal/cyclical observations over time), random fluctuations with no discernable patterns, and autocorrelation (*i.e.*, the extent to which data collected close together in time are correlated with each other) [27].

#### Sampling and population

In ITS studies, sample size calculations are related to the estimation of the number of observations or time points at which data will be collected. According to Ramsey *et al.*'s quality criteria for ITS designs, at least 10 pre- and 10 post-data points would be needed to reach at least 80% power to detect a change (if the autocorrelation is >0.4) [27]. Since the current study is a pilot, it is not known what the autocorrelation might be or what effect size the intervention is likely to produce. We therefore decided to use a relatively large number of data points to ensure that any trend or seasonal differences can be detected: 52 data points per site (where one data point = two-week segment)--26 data points before the introduction of the intervention (equivalent to 12 months' worth of two-week segments) and 26 data points after the introduction of the intervention (two-week segments for 12 months). Participants will be family physicians practicing in a solo or group practice within the Hamilton FHT and their patients at risk for osteoporosis selected according to gender- and age-eligibility criteria (*i.e.*, women ≥50 years of age, men ≥65 years of age).

#### Outcomes

Primary outcomes will be the initiation of appropriate osteoporosis investigations (*i.e.*, BMD testing) and treatment (*e.g.*, bisphosphonates, nutritional supplements such as calcium and vitamin D) during a patient visit. "Appropriate" osteoporosis management is defined as the recommendations outlined in current clinical practice guidelines from Osteoporosis Canada [12] and is represented by a disease-management algorithm programmed into the osteoporosis tool. Secondary outcomes will include fractures, the reason for the visit, number of patients who successfully complete the RAQ (defined as an electronic log generated by the tablet PC from patient-initiated RAQs), the mean time for patients to complete the RAQ, and the mapping of an osteoporosis care model for patients who will complete the RAQ (*i.e.*, documentation of what physicians do during the visit and subsequent visits with patients). Chart review will also consist of extraction of site-specific data, including the number of age-eligible patients/practice who had at least one visit during the intervention period, the number of patients who are at risk for osteoporosis, and the mean number of patients who were seen by their family physician within a two-week segment.

#### Unit of analysis and data collection

Our unit of analysis will be based on the multiple baseline assessment of individual family practice sites rather than a single group of participants being tested repeatedly before and after the introduction of the intervention. The data set at each time point will consist of patient charts, which will represent the "episode of care" used to extract outcome data for the study. To ensure the completeness and validity of the data set at each time point, we will apply the quality-control criteria of ITS designs by Ramsay *et al.*, which recommend that 80%-100% of the total number of episodes of care (*i.e.*, patient charts) be used in the data collection [27].

Once the intervention is implemented, data will be collected on all outcomes from electronic patient records (*i.e.*, PracticeSolutions^®^) and the touch-screen tablet PCs. The purpose of the pre-intervention chart review will be to establish a stable baseline of standard practice for each site. Visit-specific data (*e.g.*, initiation of osteoporosis disease management, reason for visit) will be collected bimonthly at each site by two researchers (MK and CM). To minimize the introduction of contamination that could bias results during this phase, all data collection techniques, procedures, and data collection forms will be standardized. For calibration of reliability, these two researchers will extract data from 10 randomly selected patient charts in duplicate until their agreement reaches ≥80%, at which point they will abstract data independently. The two researchers will collect data from the touch-screen tablet PCs, which will automatically generate two electronic logs each time a patient completes the RAQ. The first log will outline dated/timed RAQ responses, and the other will summarize the content of the BestPROMPT sheet. These electronic logs will be matched against patient-visit chart data to verify the use of the tool during the visit and to map any actions taken by the physician in response to the use of the RAQ.

#### Analysis

Results of this ITS study will focus on the impact of the intervention on the time series, which will be tested by comparing pre- and postintervention segments of the time series to estimate the magnitude and form of the impact. "Impact" on practice will be defined according to the level of change that is observed between the baseline and postintervention periods of the study. The impact of the tool for changing practice and satisfaction with the tool will also be analyzed across the three sites. Aggregated data from each two-week segment period on primary outcomes will be analyzed using the autoregressive integrated moving average (ARIMA) approach and time-series regression models [28,29]. We hypothesize that the 26 time-point baseline assessment of practice will show no pre-intervention trend. The ARIMA approach will estimate the extent to which a significant level of change occurs between the pre-intervention and postintervention phases of the study. The multiple measurement points are necessary for the ARIMA analysis to distinguish between treatment effects and secular trends. The advantage of using the ARIMA approach for analysis is that it accounts for the three major sources of noise that may confound the analysis: trend, seasonality, and random error [28,29]. Secondary outcomes and logs of patient-initiated data from tablet PCs will be analyzed using frequency analysis of site-specific data, descriptive and inferential statistics to calculate proportions and time to completion of the RAQ (*e.g.*, means with standard deviations), and independent-sample *t*-tests or analysis of variance (ANOVA) for group comparisons (*e.g.*, differences between sites for outcomes).

### Phase 3: qualitative postintervention follow-up study

After the 12-month intervention phase, we will conduct focus groups and interviews with participants of the pilot study (family physicians, nurse practitioners, and clinic staff from each site). The objectives of this study will be to better understand participants' experiences with and perceived utility of the tool, readiness to adopt the tool at the point of care, and satisfaction with its implementation and use in practice. This information will inform sustained use of the tool.

## Methods

Focus groups and interviews will be conducted to provoke an informal discussion about participants' experiences and satisfaction with the osteoporosis tool and to find out their readiness to sustain the tool in their practice. Questions will include participants' perceptions on barriers and facilitators to using the osteoporosis tool at the point of care, how the tool functioned in practice, whether they plan to continue using the tool in their practice, their perceptions of the tool's impact on their practice workflow, and any suggestions for improving the tool. To minimize the occurrence of "history" (a threat to internal validity where some other influential event may happen during the intervention), we will design an accompanying questionnaire to capture all clinical practice-related activities done by physicians (*e.g.*, continuing medical education [CME] activities) during the study that might account for changes between baseline and postintervention observations. We will also incorporate participant- and site-specific demographic questions and relevant items from the four-point BARRIERS scale, which can be used to assess barriers to research utilization [30].

### Analysis

Interviews and accompanying questionnaires will be quantitatively and qualitatively analyzed. Interview sessions will be audiotaped and transcribed verbatim. Data collection and qualitative content analyses will be guided by the constant comparative method of grounded theory methodology [31]. Two investigators will independently develop a coding scheme by identifying, classifying, and labelling the primary patterns in the content. Inter-coder reliability will be assessed using Kappa statistics, and any disagreements will be resolved by consensus. Data will be coded from transcripts using a process of open, axial, and selective coding [31,32] using NVivo 8 software (QSR International, Cambridge, MA, USA). During open coding, the constant comparative approach will be used to group the codes into categories and identify themes. Quantitative analysis of accompanying questionnaire data will be analyzed using analysis of variance for continuous variables (*e.g.*, Likert-type questions), chi-square tests for dichotomous variables (*e.g.*, yes/no-type questions), and content analysis for open-ended questions.

## Discussion

The osteoporosis tool has the potential to impact clinical care and to make several contributions to the development of complex, chronic disease interventions. The clinical goal of the osteoporosis tool was to bridge the gap between current and best practice in osteoporosis disease management. The three-phased evaluation study will address this goal and illustrate how its rigorous development can lead to meeting the many challenges to developing complex interventions. Without careful consideration of system design, function, and end-user perspectives, these interventions can fail [33]. If information technology systems such as the osteoporosis tool are integrated without evaluating how they might impact end users or their existing workflow, they have the potential to be ineffective, function poorly, and result in medical or technology-induced errors [34-36]. To meet the specific needs of physicians, customization of information technology systems such as the osteoporosis tool need to match and support the workflow.

We anticipate that the osteoporosis tool will benefit both physicians and patients. This benefit may include an increased awareness for patients about osteoporosis and its associated risks, the availability of relevant information about what they can do about these risks, and the opportunity to discuss this information and a management plan with their family physician at the point of care. We believe that this component is an important step toward improved self-management.

Self-management strategies can help patients manage their medical conditions and provide patients with information, skills, and the confidence (self-efficacy) to deal with their illness [37]. Moreover, patient self-management may facilitate the sustainability of an intervention by alleviating resource burdens that might be needed to maintain the ongoing use of the tool; for example, a study found that a falls-and-fractures prevention strategy in a family practice unit delivered by clinic nurses was effective, but it could not be sustained beyond the study period [38]. We have planned for the equipment (touch-screen tablets, printers, etc.) to remain at the evaluation sites permanently so that patients and physicians can continue to benefit from the tool beyond the study period if they choose to.

Self-management is becoming increasingly important for the development of chronic disease-management interventions because seniors are becoming the fastest-growing population group [39]. This is expected to increase the prevalence of chronic diseases [40] and increase the awareness and the need for patient self-care to support chronic disease management [40,41]. As a result, there is a shift toward a new patient-physician relationship for chronic disease management, where patients are becoming their own caregivers and healthcare professionals act as consultants to support their patients in this role [42,43].

### Potential limitations

Our study has several potential limitations. The ITS methodology, which was chosen for the pilot evaluation of the osteoporosis disease-management tool, is susceptible to several potential threats to internal validity. In general, we designed our methodology according to the ITS quality criteria recommended by Ramsay *et al. *to help overcome these threats and to rule out any alternative explanations of our findings [27]. Instrumentation (a threat to internal validity that could occur if the measurement method changes during the intervention and evaluation period) is a common threat in medical record data research, particularly in a multisite study. To overcome this problem, we will ensure that our databases, recording systems, observers, and outcome-measure instruments remain consistent, and they will be monitored closely for any changes that might occur over the course of the study. History is another potential threat because a change in clinical practice independent of the introduction of the intervention may occur from the influence or participation of other events and activities during the study period. For healthcare professionals and physicians in particular, these include continuing professional development activities (*e.g.*, participation in CME activities such as didactic lectures, small-group workshops, and attendance at conferences). To address this potential threat, we will collect information on continuing professional development activities using the demographic/evaluation questionnaire, which will incorporate questions targeted specifically for capturing clinical practice-related activities and events that might account for changes between baseline and postintervention observations.

### Control of the implementation of the intervention

We anticipate that once the intervention is implemented, there will be an increase trend toward optimal practice according to guidelines. This sudden rise will likely occur from the implementation process rather than the intervention itself. We therefore chose a greater number of data points (which is also equal to the data points in the baseline assessment phase) to help neutralize the initial impact of the implementation and allow the true impact of the intervention to emerge.

### Instability

Although the ITS design is susceptible to fluctuating trends and cycles, most of these unpredictable elements can be controlled statistically. We will use the ARIMA approach to analyze our data to control for the effects of variability. Additionally, we will also ensure that any variability that may occur will not be due to unreliability of the measurements (*i.e.*, outcomes will be measured objectively and assessed blindly). Lastly, the ITS methodology largely limits the generalizability of its findings [44]. However, the ITS design is a useful and pragmatic tool, particularly for pilot studies where initial evaluations of interventions and their refinement need to be done before the testing of the tool on a wider scale is justified. Furthermore, results from ITS studies can serve to inform the investigation of mediating factors (for example, if the intervention is found to be more effective in one site but not in another) as well as more extensive tests of their replicability in a randomized controlled trial.

## Competing interests

The authors declare that they have no competing interests.

## Authors' contributions

All authors participated in the design of the study. MK drafted the manuscript, and all authors read and approved the final manuscript.
